# Comprehensive Structural, Chemical, and Optical Characterization of Cu_2_ZnSnS_4_ Films on Kapton Using the Automated Successive Ionic Layer Adsorption and Reaction Method

**DOI:** 10.3390/nano15020085

**Published:** 2025-01-08

**Authors:** Perla J. Vázquez-González, Martha L. Paniagua-Chávez, Lizette A. Zebadua-Chavarria, Rafael Mota-Grajales, C. A. Meza-Avendaño, Enrique Campos-González, A. Escobosa-Echavarría, Yaoqiao Hu, Aldo E. Pérez-Ramos, Manuel-Matuz, Carlos A. Hernández-Gutiérrez

**Affiliations:** 1 Tecnológico Nacional de México Campus Tuxtla, Carretera Panamericana Km 1080, Tuxtla Gutiérrez C.P. 29050, Mexicorafael.mg@tuxtla.tecnm.mx (R.M.-G.); 2 Programa de Nanociencias y Nanotecnología, Centro de Investigación y de Estudios Avanzados del Instituto Politécnico Nacional, Av. Instituto Politécnico Nacional 2508, México City C.P. 07360, Mexico; 3 Instituto de Investigación e Innovación en Energías Renovables, Universidad de Ciencias y Artes de Chiapas, Libramiento Norte 1150 Col. Lajas Maciel, Tuxtla Gutiérrez C.P. 29039, Mexico; carlos.meza@unicach.mx; 4 Departamento de Física, Instituto Nacional de Investigaciones Nucleares, Carretera México-Toluca s/n, La Marquesa, Ocoyoacac C.P. 52750, Mexico; enrique.campos.conacyt@inin.gob.mx; 5 Departamento Ingeniería Eléctrica—SEES, Cinvestav-IPN, México C.P. 07360, Mexico; escobosa@cinvestav.mx; 6 Department of Materials Science and Engineering, The University of Texas at Dallas, Richardson, TX 75080, USA; yaoqiao.hu@utdallas.edu; 7 Tecnológico Nacional de Mexico/I.T. de Oaxaca, Calz. Tecnológico No. 125, Oaxaca C.P. 68030, Mexico; dr.aldo@itoaxaca.edu.mx; 8 Tecnológico Nacional de México Campus Tapachula, Carretera a Puerto Madero Km. 2, Centro, Tapachula de Córdova y Ordoñez C.P. 30700, Mexico; mjmatuz@tapachula.tecnm.mx

**Keywords:** SILAR, CZTS, flexible, solar cells

## Abstract

This study provides a comprehensive structural, chemical, and optical characterization of CZTS thin films deposited on flexible Kapton substrates via the Successive Ionic Layer Adsorption and Reaction (SILAR) method. The investigation explored the effects of varying deposition cycles (40, 60, 70, and 80) and annealing treatments on the films. An X-ray diffraction (XRD) analysis demonstrated enhanced crystallinity and phase purity, particularly in films deposited with 70 cycles. These films exhibited a notable reduction in secondary phases in the as-deposited state, with further improvements observed after annealing at 400 °C and 450 °C in a sulfur atmosphere. A pole figure analysis indicates a decrease in texture disorder with annealing, suggesting improved crystalline orientation at higher temperatures. Field emission scanning electron microscopy (FE-SEM) showed enhancements in surface morphology, with increased grain size and uniformity post-annealing. Chemical uniformity was confirmed through Secondary Ion Mass Spectrometry (SIMS), Energy-Dispersive Spectroscopy (EDS), and X-ray Photoelectron Spectroscopy (XPS). XPS revealed the presence of CZTS phases alongside oxidized phases. Annealing effectively reduced secondary phases, such as ZnO, SnO_2_, CuO, and SO_2_, enhancing the CZTS phase. An optical analysis demonstrated that annealing at 200 °C in an air atmosphere reduced the band gap from 1.53 eV to 1.38 eV. In contrast, annealing at 400 °C and 450 °C in a sulfur atmosphere increased the band gap to 1.59 eV and 1.63 eV, respectively. The films exhibited p-type conductivity, as inferred from a valence band structure analysis. Density Functional Theory (DFT) calculations provided insights into the observed band gap variations, further substantiating the findings.

## 1. Introduction

As renewable energy sources, solar cells employ semiconductors with specific optoelectronic properties. Silicon is the most widely used semiconductor in solar cells, primarily due to its well-established technology. However, silicon’s indirect band gap produces a low absorption coefficient, requiring thicker devices (around 100 µm). Additionally, silicon-based processes involve costly techniques such as ion implantation and high-temperature treatments [1]. Conversely, thin-film solar cells, particularly those made from CdTe and CIGS, offer a more cost-effective alternative. However, the associated toxicity of CdTe, the low abundance, and the high cost of indium in CIGS cells present challenges [2,3,4,5,6]. Research has focused on new materials with direct band gaps, high absorption coefficients, and abundant elements to enhance the cost-efficiency ratio of solar cells. CZTS stands out as a promising candidate with its optimal band gap according to the Shockley–Queisser limit and high absorption coefficient (approximately 10^4^ cm^−1^) [7]. Additionally, semiconductors with a quaternary kesterite tetrahedral structure, particularly Cu_2_ZnSnS_4_ (CZTS), Cu_2_ZnSnSe_4_ (CZTSe_4_), and Cu_2_ZnSn(S_x_,Se_1−x_)_4_, are increasingly being explored as active layers for economical thin-film solar cell applications [8]. Various methods to perform kesterite thin films have been investigated, including vacuum-based physical deposition and non-vacuum chemical methods. While vacuum-based methods such as sputtering and co-evaporation are common, they are less suitable for industrial-scale applications due to high costs and energy consumption. In contrast, non-vacuum methods offer scalability and economic benefits, producing uniform films without vacuum environments or high-temperature processes, simplifying preparation. Non-vacuum techniques have several advantages for thin film deposition, such as creating homogenous, high-quality crystals over large areas without high temperatures or vacuum. Many materials, including CZTS, have been successfully deposited using these methods, showing promise for solar cell applications. Advances in automation have led to the development of electromechanical systems for controlling non-vacuum processes, like dip-coating, spray pyrolysis, and Sol–Gel machines, which are now enhanced with microcontrollers for precision and cost reduction. Among them, the SILAR method is notable for its precise control and consistent results in terms of thickness. Therefore, the non-vacuum low-cost SILAR technique has been used to deposit CZTS thin films for solar cell applications [9,10,11,12]. Regarding deposition on flexible substrates, Kakherskyi et al. reported the use of a spray technique to deposit CZTS nanoparticles on polyamide [13]. Dilara Gokcen Buldu et al. investigated the influence of sulfurization temperature on Cu_2_ZnSnS_4_ (CZTS) thin films deposited on chemically etched, flexible titanium (Ti) substrates [14]. Similarly, Yaqun Liu et al. utilized a two-step process involving magnetron sputtering of Cu-Zn-Sn precursors onto molybdenum foils, followed by sulfurization, to deposit CZTS thin films on flexible substrates [15]. Qichen Zhao et al. also explored flexible titanium substrates for solar cell applications, employing a two-step method comprising DC sputtering and subsequent sulfurization. Their process included polishing Ti foils, sputtering a Mo back electrode, and spin-coating with various metal acetates, resulting in high-quality CZTS and CZTS absorber films post-sulfurization [16]. Based on our comprehensive literature review, there are no reports on the direct deposition of CZTS on polyamide Kapton substrates using the SILAR technique. Furthermore, previous studies lack complete structural characterization (XRD and texture analysis) and chemical analyses (SIMS, EDS, and XPS). Therefore, the primary objective of this work is to thoroughly report the structural, chemical, and optical properties of CZTS films deposited on polyamide (Kapton) substrates, focusing on their potential for flexible applications.

## 2. Experimental Details

The SILAR system was designed and constructed with precision mechanical components using the V-Slot Modular Building System from OPENBUILDS Mexico. This setup ensured smooth linear motion and high reliability. The *X*-axis module, equipped with a GT2 timing pulley and belt, facilitated a linear movement of up to 1200 mm. The *Y*-axis module, integrated with a 3D robotic arm and substrate holder, employed an iron spindle for seamless sliding. The electrical and electronic circuitry featured a dual power supply configuration: a 12 Vdc/10 A supply dedicated to the stepper motors and controllers, and a 5 Vdc/5 A supply for electronic components, including Hall effect sensors, a seven-segment display, and the Raspberry Pi Pico microcontroller. The microcontroller served as the main controller for the SILAR robot, ensuring precise execution of the deposition process. The SILAR process consists of four fundamental steps: adsorption of cationic precursors, rinsing of unbound cations, reaction with anionic precursors, and rinsing away non-reactive anions to minimize deposition defects.

**Figure 1:**
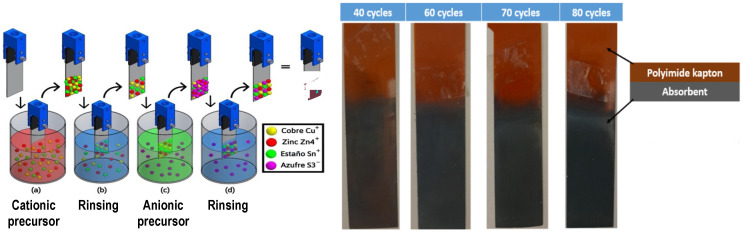
The chemical process of CZTS deposition by SILAR method, and the deposited CZTS samples over the Kapton as a function of the number of cycles.

CZTS thin films were deposited on flexible Kapton substrates using cationic and anionic precursor solutions. The cationic solution contained 0.015 M CuCl_2_, 0.075 M ZnSO_4_, and 0.01 M SnCl_2_, while the anionic solution contained 0.15 M Na_2_S. Deposition parameters were carefully optimized to achieve high-quality film formation, including dipping time, number of cycles, dipping temperature, rinsing temperature, and horizontal axis speed. As shown in Figure 1, the number of deposition cycles directly influenced the film thickness and light absorption, with darker films observed as the cycle count increased from 20 to 60. Following deposition, the films were annealed under two different conditions: 200 °C in an air atmosphere for 60 min, and 400 °C and 450 °C in a sulfur atmosphere for 20 min each. These annealing steps aim to enhance crystallinity, improve sulfur incorporation, and reduce sulfur escape.

### Characterization Techniques

Structural Analysis: X-ray diffraction (XRD) measurements were performed using a Rigaku Smart Lab system equipped with a 9 kW rotating copper anode (λ = 1.5406 Å). The scans were conducted in the 2θ range of 20–80° at a grazing incidence angle of 1°. Pole figure analyses were conducted to evaluate crystalline orientation. The surface morphology and composition were analyzed using an SEM-EDS JEOL JSM–7401F microscope with an acceleration voltage of 3–10 kV.

SIMS: Secondary Ion Mass Spectrometry employed a double beam analysis regime. A cesium ion beam (500 eV, 60 nA) raster-scanned a 500 × 500 μm^2^ area, and a pulsed Bi3+ ion beam analyzed a central 150 × 150 μm^2^ region. Depth profiling was conducted using a Dektak XT profiler. High-resolution XPS analysis was conducted using a Thermo K-Alpha system with an Al Kα X-ray source (1486 eV). The spectra were calibrated to the adventitious carbon peak (C1s, 284.8 eV) and deconvoluted using Shirley background subtraction. Scofield’s relative sensitivity factors were used for quantification.

An Optical Analysis was performed using an Ocean Optics ISP-R-GT integrating sphere cavity. The total reflection percentage was recorded with a UV–Vis spectrometer (Ocean Optics USB 4000). The Kubelka–Munk method was applied to extract the band gap energy from the reflectance data.

Density Functional Theory (DFT) calculations were performed by using Vienna ab initio Simulation Package (VASP)1-3. Projector augmented wave (PAW)4,5 [17,18] pseudopotentials were used. The generalized gradient approximation (GGA)6 [19] of a Perdew−Burke−Ernzerhof (PBE)7 [20]-type functional was employed to describe electronic exchange and correlation interaction. For all calculations, a cutoff energy of 420 eV was used for plane wave basis set expansion. Brillouin zone k-point sampling used the Gamma-centered Monkhorst−Pack scheme. Vacancy defects were created by removing one constituting atom from the unit cell. Geometric structures were then optimized using the conjugate method (CG) with the convergence criterion being the force on each atom less than 0.01 eV/Å. Electronic minimization was achieved through a blocked Davidson scheme with the stopping condition being the energy difference between two adjacent iterations less than 5–10 eV [21,22,23].

## 3. Results and Discussions

### CZTS over a Flexible Substrate

**Figure 2:**
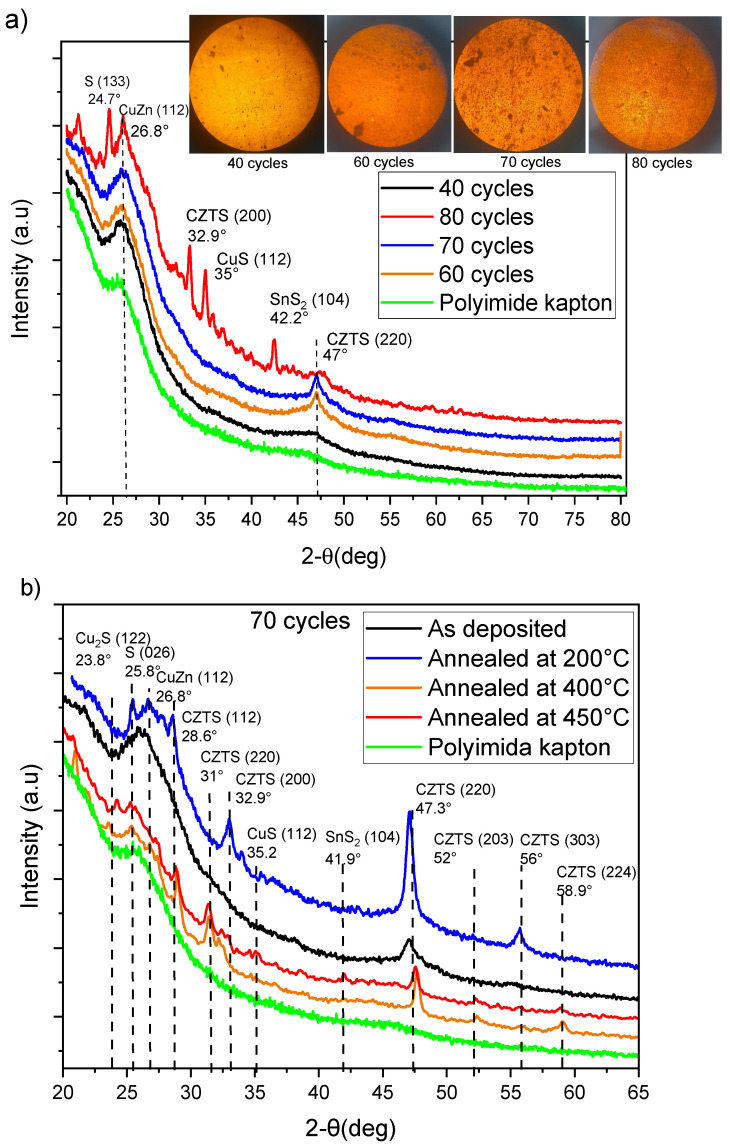
XRD patterns of (**a**) the as-deposited samples as a function of deposition cycles, and (**b**) the samples deposited with 70 cycles and those annealed at 200 °C, 400 °C, and 450 °C.

The structural properties of CZTS films deposited on Kapton substrates were investigated using X-ray diffraction (XRD). The Kapton substrate served as a reference, showing broad and weak signals characteristic of amorphous materials, which contributed mainly to the background without obstructing the detection of crystalline CZTS phases. A series of samples prepared with 40, 60, 70, and 80 deposition cycles were analyzed to optimize the deposition parameters. The X-ray diffraction pattern of the as-deposited 80-cycle sample showed a combination of amorphous signals and CZTS diffractions at angles of 33° and 47°. These angles correspond to the (200) and (220) crystalline planes of the CZTS kesterite [24,25]. In addition, diffraction peaks at 26° and 42° were observed, associated with CuZn in the (112) and (020) planes, respectively. Furthermore, A peak at 35° was attributed to the CuO (002) plane [25]. In contrast, samples with fewer than 80 cycles showed a diffraction peak corresponding to the CZTS (220) plane at 47° [25,26,27], with the 70-cycle sample yielding the most favorable results. Additionally, microscopic images were captured to evaluate the homogeneity and porosity of the samples, as shown in the inset of Figure 2a. Subsequently, the samples were annealed at 200 °C for 60 min in an air atmosphere to enhance their structural properties. Further annealing processes were conducted at 400 °C and 450 °C in a sulfur atmosphere for 20 min each, as shown in Figure 2b. After annealing, new diffraction peaks were observed at 28°, 32°, and 55°, corresponding to the CZTS planes (112), (200), and (312), respectively, along with the characteristic CZTS reflection at 47° corresponding to the (220) plane [25,28]. Peaks at 25° and 26° are attributed to secondary phases such as sulfur S and CuZn present in the as-deposited films. Upon annealing at 400 °C and 450 °C, the intensity of the CuZn peak decreases significantly, indicating a reduction in this secondary phase. In contrast, additional CZTS peaks emerge at 31° and 59° [28], suggesting improved crystallinity and phase purity of the CZTS structure. A slight shift toward higher diffraction angles near the CZTS (220) plane (47°) was observed for samples annealed at 400 °C and 450 °C, which can be attributed to an increased sulfur content within the CZTS lattice. Additionally, the results indicate that annealing improves the crystallinity of CZTS thin films, with 400 °C emerging as an optimal temperature for reducing secondary phases and promoting the formation of well-defined CZTS planes.

**Figure 3:**
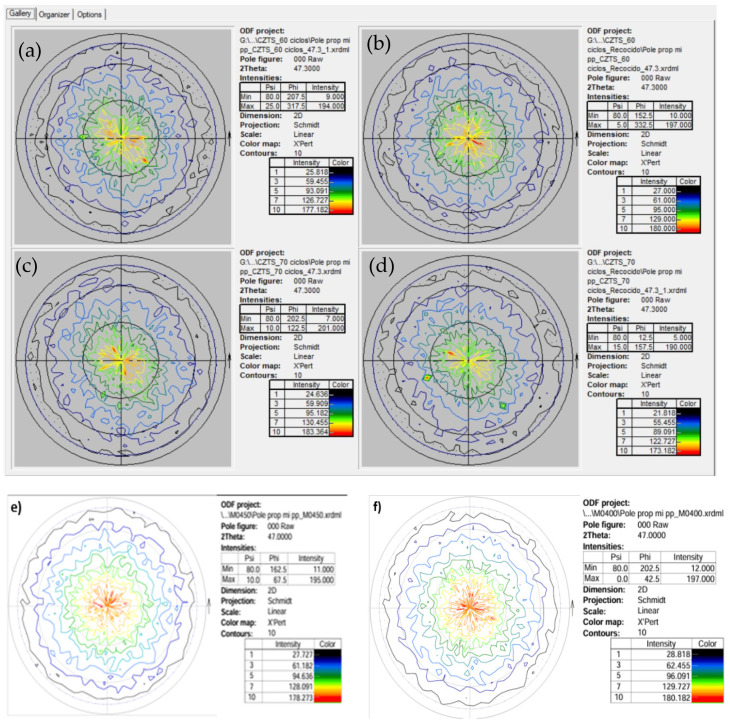
Texture analysis of the studied samples: (**a**) 60 cycles as-deposited, (**b**) 60 cycles after annealing, (**c**) 70 cycles as-deposited, (**d**) 70 cycles after annealing at 200 °C, (**e**) 70 cycles after annealing at 450 °C, (**f**) 70 cycles after annealing at 400 °C.

Furthermore, the XRD pole figure analysis for the as-deposited and annealed samples is shown in Figure 3. This analysis reveals no significant changes in texture, although a slight improvement in texture uniformity is observed in the post-annealed 70-cycle sample. The as-deposited 60-cycle sample (Figure 3a) exhibits diffuse and poorly defined contours, suggesting a low degree of crystalline orientation and textural uniformity. This indicates that 60 cycles of deposition are insufficient to promote a well-defined crystalline texture. Annealing the 60-cycle sample (Figure 3b) shows a slight improvement in texture. In contrast, the 70-cycle as-deposited sample (Figure 3c) reveals a notable improvement in crystalline texture, with clearer and more concentrated contours. This indicates that increasing the number of deposition cycles promotes better crystalline orientation and reduces textural anisotropy. When the 70-cycle sample is annealed at 200 °C (Figure 3d), the texture shows further refinement. A significant improvement in texture is observed when the 70-cycle samples are annealed at 400 °C and 450 °C (Figure 3e,f). Annealing at 450 °C results in well-defined and concentrated contours, indicating enhanced crystalline alignment and grain growth. Similarly, annealing at 400 °C yields comparable results. Both temperatures facilitate the reorganization and growth of grains, leading to more uniform textures with reduced anisotropy.

**Figure 4:**
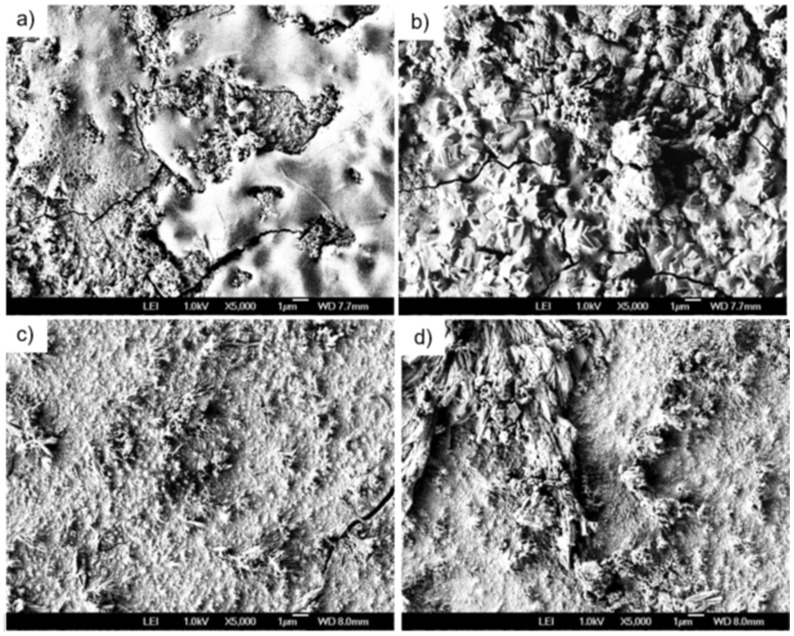
F-SEM analysis of the studied samples: (**a**) 60 cycles as-deposited, (**b**) 60 cycles after annealing, (**c**) 70 cycles as-deposited, and (**d**) 70 cycles after annealing at 200 °C.

**Figure 5:**
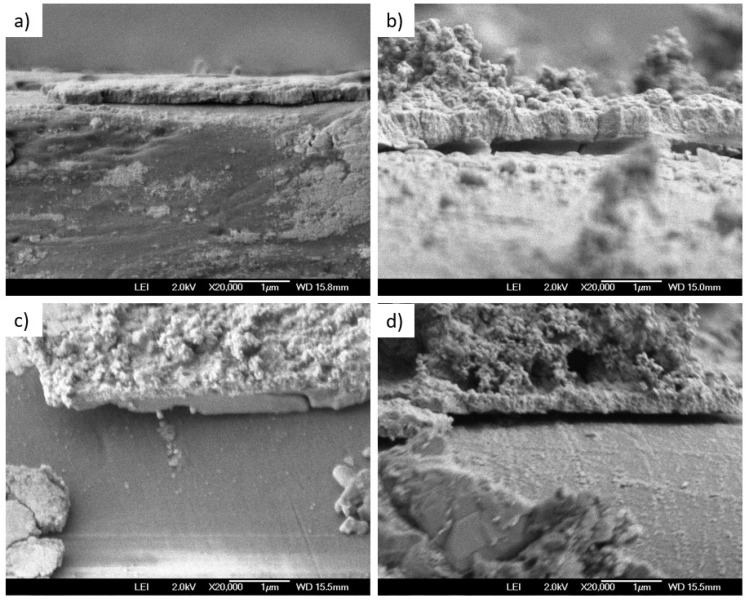
F-SEM cross-section analysis of the studied samples, (**a**) 60 cycles as-deposited, (**b**) 60 cycles after annealing, (**c**) 70 cycles as-deposited, and (**d**) 70 cycles after annealing at 200 °C.

Surface morphology was analyzed using F-SEM. The SEM images in Figure 4 depict the surface morphology of samples processed with different cycles and post-deposition annealing treatments. The 60-cycle as-deposited sample shows a relatively heterogeneous surface with areas of smooth texture interspersed with more granular and rough regions, suggesting variability in the crystalline structure or composition. The 70-cycle as-deposited sample exhibits a more uniform granular morphology, with clearly defined, faceted crystalline structures. This suggests a higher degree of crystallization compared to the 60-cycle sample in good correlation with XRD. The 60-cycle sample, after annealing, presents a denser and more homogeneous surface. Annealing appears to have smoothed out the surface irregularities, leading to a more consistent grain size across the sample. The 70-cycle sample, post-annealing, displays an even more pronounced transformation. The annealing process seems to have caused significant coalescence of the grains, leading to larger crystalline structures. So, annealing improves the surface smoothness and crystalline quality in both samples, with the 70-cycle samples showing more pronounced grain growth. In addition, the cross-sectional F-SEM images shown in Figure 5 provide a detailed view of the morphological variations among the samples, reflecting the influence of different deposition and post-treatment conditions. The 60-cycle as-deposited sample exhibits a layered structure with visible gaps and signs of peeling. In contrast, the 60-cycle post-annealing sample shows a rougher surface with increased texturing. The 70-cycles as-deposited samples present a notable improvement with a smoother, more consistent surface, suggesting that modifications in the deposition conditions have positively impacted the film’s uniformity and structural coalescence. Finally, the 70-cycle post-annealing samples displayed the most refined morphology with a dense, uniform structure and minimal surface defects. Furthermore, grains are observed in good correlation with structural characterization. However, it is worth mentioning that submicron-size grains are observed, indicating that the annealing could be improved for future work to enhance the grain size.

**Figure 6:**
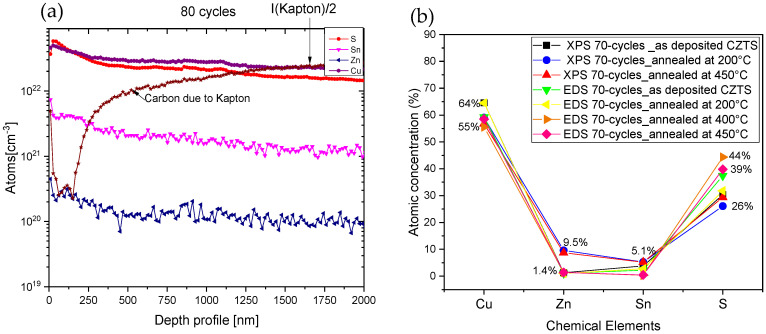
(**a**) SIMS depth profile of the CZTS deposited on Kapton, (**b**) EDS and XPS quantification results for the analyzed samples.

The chemical analysis was performed by Secondary Ion Mass Spectrometry (SIMS), Energy-Dispersive Spectroscopy (EDS), and X-ray Photoelectron Spectroscopy (XPS). SIMS was carried out to perform a chemical depth profile, revealing a uniform chemical composition throughout the film, as shown in Figure 6a. The SIMS analysis corroborated the film’s porosity, consistent with observations from optical microscopy (illustrated in the inset of Figure 2). From the SIMS analysis, the film thickness is approximately 1.6 µm, which is suitable for solar cell applications due to the CZTS’s high absorption coefficient of 10^4^ cm^−1^. This implies that a thickness of approximately 1 µm is suitable to absorb most of the wavelengths near the band gap edge. The EDS results shown in Figure 6b corroborate the SIMS analysis, revealing a high concentration of copper, while tin and zinc appear in lower quantities.

**Figure 7:**
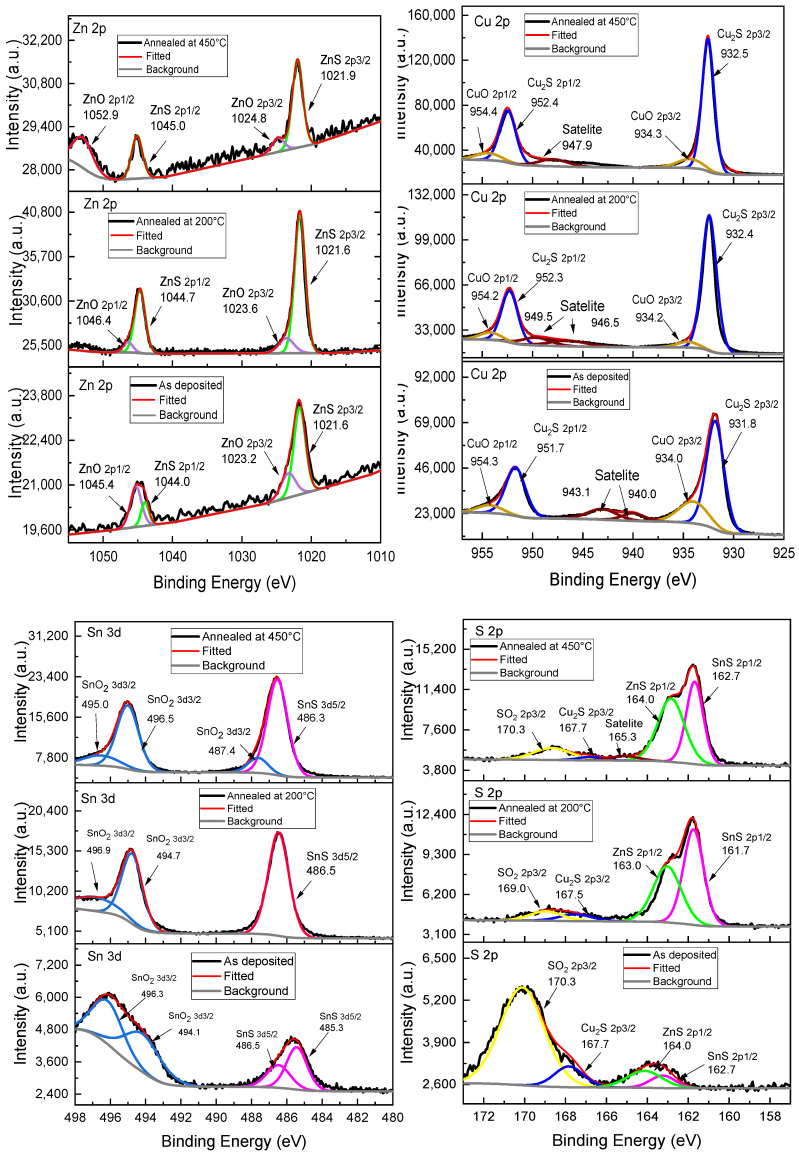

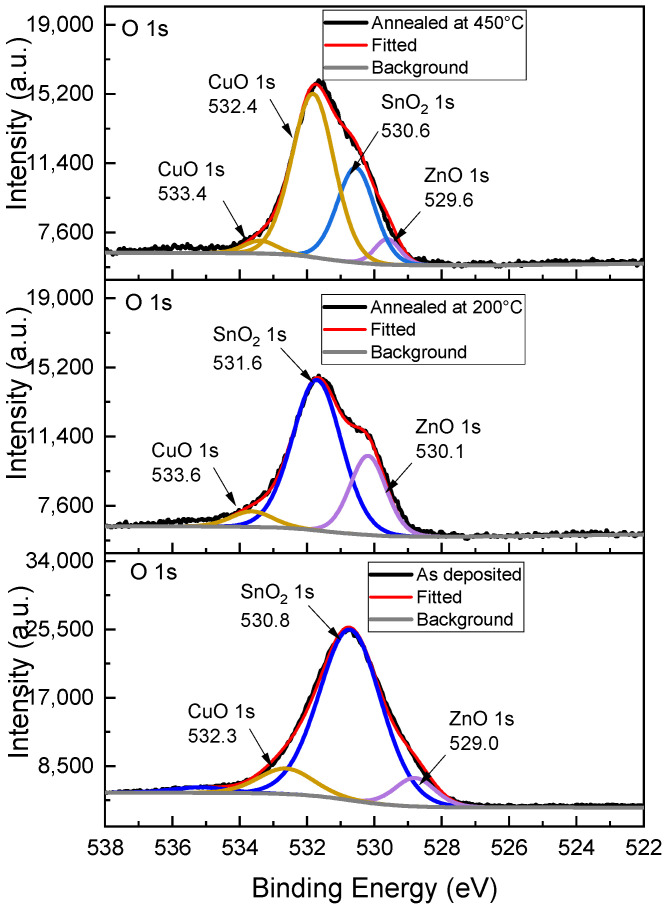
XPS analysis of CZTS elements for as-deposited films and films annealed at 200 °C and 450 °C.

A high-resolution X-ray Photoelectron Spectroscopy (XPS) analysis was carried out on both as-deposited and annealed samples to explore the chemical states within the bulk material. The XPS characterizations for the as-deposited, annealed at 200 °C in an air atmosphere, and annealed at 450 °C in sulfur atmosphere samples are shown in Figure 7. The Cu 2p photoelectron emission predominantly indicates the presence of Cu_2_S, with CuO formation [29,30,31]. The samples annealed revealed a significant reduction of CuO, favoring the formation of a purer phase of Cu_2_S. The Zn 2p spectrum detected primarily ZnS [29,30] and the presence of ZnO [32]. The sample annealed at 200 °C reduced the presence of oxygen in the film and increased the ZnS signal. The sample annealed at 450 °C displayed a clear reduction in the oxygen element, along with a ZnS signal. Sn 3d signals showed contributions from SnO_2_ [29,32] and SnS [30,33]. SnO_2_ is present in the as-deposited sample, and the SnS signal is notably enhanced by annealing at both 200 °C and 450 °C. Additionally, the S 2p spectrum revealed the presence of several bounds, including SO_2_, Cu_2_S [30,34], ZnS [34,35], and SnS [33]. During annealing, a reduction in SO_2_ and Cu_2_S 2p3/2 signals is observed, enhancing the signals of ZnS and SnS, which suggests a greater formation of these sulfurous phases during annealing. The oxygen 1s spectrum confirmed the presence of CuO_2_ [32], SnO_2_ [33], and ZnO [35,36] indicating a complex combination of oxides influencing the film’s composition. The comparison of XPS results for samples annealed at 200 °C and 450 °C indicates that annealing at 450 °C improves sulfur retention, as the sulfur loss is significantly reduced compared to the sample annealed at 200 °C. Figure 6b quantifies the elemental composition necessary to form CZTS. However, XPS indicates the presence of oxide phases within the films. The CZTS Bragg reflection is attributed to crystals of pure CZTS phases, suggesting that while some regions of the film consist of pure CZTS, the overall composition may not be fully stoichiometric. So, after the deconvolution analysis, the atomic concentrations indicate a nonstoichiometric film with an excess of Cu and a deficit of S. For future investigations, it would be beneficial to explore adjustments in the Cu content of the precursor formula and increase the amount of sulfur in the annealing atmosphere to improve the stoichiometry of the film.

**Figure 8:**
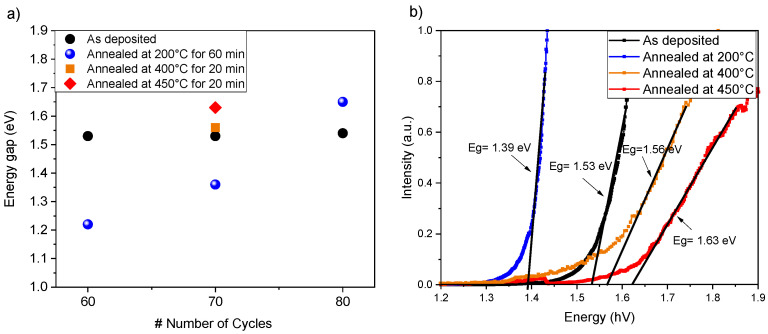
(**a**) Band gap energy measurements of the studied samples, and (**b**) band gap extraction graph using the Kubelka–Munk method for the studied samples.

The optical characterization of the samples was performed using UV-VIS, as summarized in Figure 8. The band gap was determined using the Kubelka–Munk method, revealing an initial band gap of approximately 1.53 eV for the as-deposited samples, independent of the number of SILAR cycles. Annealing at 200 °C in an air atmosphere decreased the band gap to 1.38 eV for samples deposited with 60 and 70 cycles. In contrast, a slight increase in the band gap was observed for the 80-cycle samples, attributed to the formation of multiple phases within the films. In comparison, annealing at 400 °C and 450 °C in a sulfur atmosphere led to further changes. The sample annealed at 400 °C showed a slight increase in the band gap to 1.58 eV, indicating improved sulfur incorporation, whereas the sample annealed at 450 °C reached a band gap of 1.63 eV, suggesting potential phase transitions or improved crystallinity. On the other hand, the band gap increase could be related to Sn reduction by further annealing, as suggested by EDS. Among all the studied samples, the 70-cycle demonstrated the most balanced properties, achieving a band gap within the optimal range and superior crystallinity, as corroborated by XRD and UV-Vis analyses.

To further understand this, the (220) plane grain size of CZTS was estimated using the Debye–Scherrer equation, as shown in Table 1 [37]. For the samples deposited with 70 cycles, the grain size increased with higher annealing temperatures. At 200 °C, the grain size was approximately 13.33 nm, growing to 18.9 nm at 400 °C and reaching 28.0 nm at 450 °C. The phase analysis by XRD indicates that the annealing process promotes an increase in nanocrystal grain size. The observed increase in the band gap may be partially explained by quantum confinement effects. To evaluate the quantum confinement effect, Equation (1) models the band gap dependence on nanocrystal size.

(1) $$E(r)=E_{Gap}+\frac{h^{2}}{8r^{2}}\left(\frac{1}{m_{e}^{*}}+\frac{1}{m_{h}^{*}}\right)-\frac{1.8e^{2}}{\varepsilon_{0}\varepsilon_{r}r}$$

where *E(r)* is the band gap energy as a function of r, the ratio of the nanocrystal; *E_Gap_* is the bulk band gap; *h* is the Planck constant; m_e_* is the electron effective mass; and m_h_* is the hole effective mass [38]. For a grain size of 28.0 nm, quantum confinement contributes to an increase of 5.5 meV, yielding a band gap of 1.521 eV. However, this effect alone cannot account for the total observed enhancement of 100 meV (resulting in a band gap of 1.63 eV). Therefore, the additional increase is attributed to stoichiometric changes in the material induced by the annealing process.

In contrast to this research, Ganesh Kumar et al. reported investigations on CZTS thin films coated using the SILAR method and annealed at 250 °C in an air atmosphere for four hours. Their study observed films with low crystal quality, multiple phases, and low-intensity XRD signals, along with a grain size of 21.9 nm. In comparison, our samples demonstrated a clear (220) CZTS plane reflection and an improved grain size of 28 nm after annealing at 450 °C for 20 min [7].

**Figure 9:**
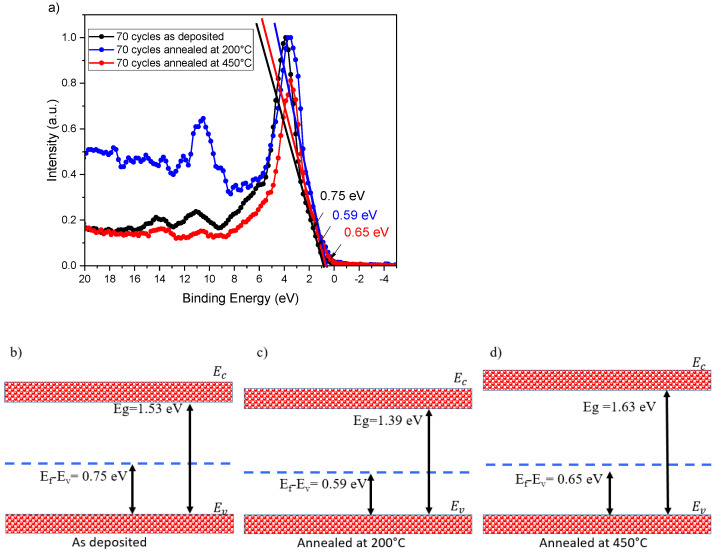
(**a**) Valence band maximum analysis for 70-cycle as-deposited and annealed samples, (**b**) band gap diagram of the as-deposited sample, (**c**) band gap diagram of the sample annealed at 200 °C, and (**d**) band gap diagram of the sample annealed at 450 °C.

Furthermore, a valence band analysis was performed for 70-cycle as-deposited and annealed samples. The valence band maximum (VBM) analysis indicates that the samples exhibit p-type conductivity. Annealing reduced the energy difference (Ef-Ev) from 0.75 to 0.59 eV. The sample annealed at 200 °C exhibited a Fermi level to valence band maximum difference of 0.59 eV, while the sample annealed at 450 °C showed a difference of 0.65 eV. Thus, a band gap estimation, complemented by a valence band analysis, was used to construct the band gap diagram shown in Figure 9b. Consequently, for applications in solar cells or photodetectors, these films have the potential to form a PN junction when paired with a well-established n-type CdS layer.

On the other hand, David B. Mitzi et al. reviewed the impact of stoichiometry on the Cu/(Zn+Sn) ratio in sputtered CZTS solar cells, highlighting that a Cu-poor, Zn-rich composition (optimal ratio ~1.2) is crucial for achieving higher efficiencies [39]. However, it is important to note that sputtering is a vacuum-based deposition technique, whereas the SILAR method employed in this study is a low-cost, non-vacuum alternative. Essential properties for efficient solar cell functionality, such as an appropriate band gap, high absorption coefficient, controlled film thickness, and p-type conductivity, were achieved in this work. The XPS analysis identified the coexistence of pure CZTS and oxide phases, indicating that the Cu/(Zn+Sn) ratio supports the formation of phases beneficial for solar cell performance. Future studies will aim to refine the film’s stoichiometry and reduce oxide content to further enhancement and suitability for photovoltaic applications.

**Figure 10:**
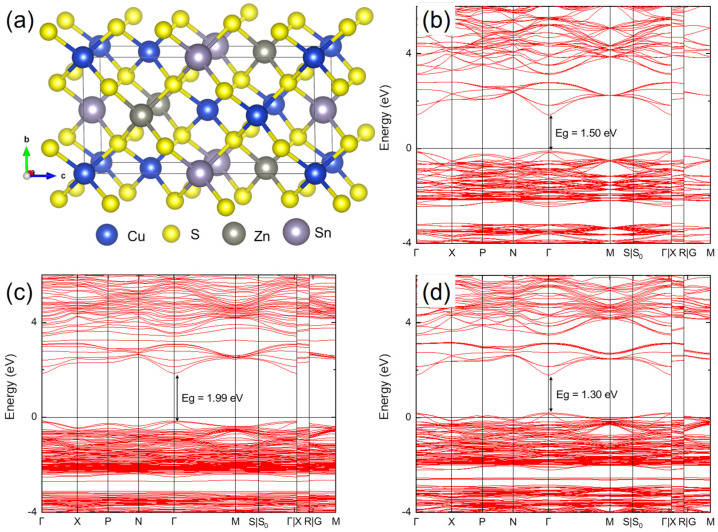
(**a**) Atomic structure of CZTS used for DFT modeling. Band structures of (**b**) pristine CZTS, (**c**) CZTS with a sulfur (S) vacancy, and (**d**) CZTS with a zinc (Zn) vacancy.

DFT calculations were performed to gain deeper insights into the origin of the band gap variation. An analysis of XPS and EDS data revealed a significant decrease in sulfur atomic concentration after annealing at 200 °C, while the zinc content increased. Figure 10 illustrates the band structure of pristine CZTS, along with the crystal structure used in this study. The CZTS crystal adopts a tetragonal system with lattice parameters a = 5.43 A° and c = 10.84 A°. Pristine CZTS exhibits a direct band gap of 1.50 eV, with the band edges located at the Γ point. Introducing a sulfur vacancy increases the band gap to 1.99 eV, while a zinc vacancy decreases it to 1.30 eV. Additionally, considering antisite defects, where Zn substitutes S, results in a band gap of 1.37 eV. From these findings, we conclude that the reduction in the band gap observed in the annealed sample at 200 °C is associated with grains where Zn concentration increases, as Zn vacancies in the as-deposited samples are partially filled during annealing. However, XPS data for the sample annealed at 450 °C reveal a similar Zn content to the sample annealed at 200 °C but with a higher sulfur content, which contradicts the Zn antisite estimations. Conversely, an EDS analysis, which focuses on elemental characterization without linking to electronic states, indicates further reductions in Zn and Sn concentrations after annealing. This observation aligns with the band gap increase being associated with sulfur vacancies that are not compensated by Zn, as its content decreases even with the slight increase in sulfur introduced by the sulfur annealing atmosphere. Therefore, based on our experimental data and DFT calculations, we conclude that the band gap variation is predominantly driven by the presence of sulfur vacancies.

## 4. Conclusions

This study demonstrates the versatility and efficacy of the Successive Ionic Layer Adsorption and Reaction (SILAR) method for depositing (CZTS) thin films on flexible Kapton substrates. A custom-designed mechatronic system provided precise control over deposition parameters, enabling the optimization of film characteristics for solar cell applications. Optical, structural, and chemical characterizations confirmed the potential of these films as absorber layers in thin-film solar cells. A structural analysis via XRD revealed that increasing the number of SILAR cycles, coupled with annealing at optimal temperatures, significantly enhanced the crystallinity and minimized secondary phases. In particular, annealing at 400 °C and 450 °C in a sulfur atmosphere improved the CZTS phase purity, highlighting the importance of these conditions for achieving superior structural properties. Chemical analyses through XPS and EDS indicate a non-stoichiometric composition, with excess Cu and a deficit of S, pointing to opportunities for refining the precursor formula and annealing atmosphere to achieve better stoichiometry. Further analysis showed that sulfur vacancies played a dominant role in the band gap variation, as confirmed by experimental data and DFT calculations. While the band gap of the films could be tuned by adjusting the deposition and annealing parameters, ranging from 1.53 eV to 1.63 eV, the influence of sulfur vacancies was critical to explain the increase in the band gap.

## Figures and Tables

**Table 1:** XRD Debye–Scherrer crystal size and energy gap E(r) calculations, considering an X-ray wavelength of λ=0.15406 nm λ=0.15406 nm.

CZTS-as Deposited					
Sample	Plane	2θ (°)	FWHM (°)	D (nm)	*E(r)*
**40 cycles**	(220)	46.7	0.22	39.3	1.511
**60 cycles**	(220)	47.06	0.54	16.0	1.565
**70 cycles**	(220)	47.04	0.48	18.1	1.551
**80 cycles**	(220)	47.54	0.14	62	1.504
**CZTS-70 Cycles Annealed**					
**Annealed**	**Plane**	**2θ (°)**	**FWHM (°)**	**D (nm)**	***E(r)***
**200 °C**	220	47.14	0.65	13.33	1.594
**400 °C**	220	47.68	0.46	18.9	1.548
**450 °C**	220	47.54	0.31	28.0	1.521

## Data Availability

The data are contained within the article.
